# Losing the Home Field Advantage When Playing Behind Closed Doors During COVID-19: Change or Chance?

**DOI:** 10.3389/fpsyg.2021.658452

**Published:** 2021-04-15

**Authors:** Yannick Hill, Nico W. Van Yperen

**Affiliations:** ^1^Department of Psychology, University of Groningen, Groningen, Netherlands; ^2^Center for Human Movement Sciences, University of Groningen, University Medical Center Groningen, Netherlands

**Keywords:** social facilitation, social support, sport performance, spectators and fans, bootstrapping analysis, randomness

## Abstract

Due to restrictions against the COVID-19 pandemic, spectators were not allowed to attend soccer matches at the end of the 2019/2020 season. Previous studies suggest that the absence of a home crowd changes the home field advantage in terms of match outcomes, offensive performance, and referee decisions. However, because of the small sample sizes, these changes may be random rather than meaningful. To test this, we created 1,000,000 randomized samples from the previous four seasons with the exact same number of matches played behind closed doors in Europe’s four most elite soccer leagues at the end of the 2019/2020 season. We found that across countries (Germany, Spain, Italy, and England), performance indices and referee decisions (except red cards) indeed changed to the detriment of the home team beyond the level of chance. However, this overall pattern could be ascribed to specific countries. Most importantly, the proportion of points won by the home teams declined significantly only in Germany, which was accompanied by a meaningful increase in (1) the proportion of goals scored by the away teams and (2) the proportion of yellow cards given to the home teams. We conclude that the home field advantage may indeed be lost when spectators are absent. However, in future studies, more detailed behavioral analyses are needed to determine the robustness and the behavioral determinants of this phenomenon across leagues and countries.

## Introduction

In a typical professional soccer match, two teams compete with each other in front of a large audience. Especially the home teams are supported and cheered on by large numbers of people in the audience often referred to as “the home team’s 12th man.” Due to the COVID-19 pandemic that started out during the 2019/2020 season, professional soccer matches took place without spectators. On May 16, 2020, the German Soccer Bundesliga became the first major league in any sport in the world to resume its season without spectators. Soon thereafter, the Spanish, English, and Italian elite divisions also resumed their competitions. This provides the opportunity to identify possible performance changes across leagues and countries, which may be primarily ascribed to the absence of the spectators.

Contemporary theories of sports performance explicitly emphasize the role of the spectators by arguing that the performance emerges from the interactions between the athletes and their environment, which includes spectators or their opponents (Davids et al., 2013; Gorman et al., 2017). However, the notion that the spectators present at a sports event can influence the performers is not a new development but has been embedded in the literature on sport and performance psychology for many years. Specifically, at the end of the 19th century, Triplett (1898) observed that racing cyclists performed faster in a competitive context (i.e., when racing against another racer) than when racing against the clock alone. In follow-up studies, he discovered that simply the presence of an audience had the same effect, a psychological principle that we now know as social facilitation (Allport, 1924; Zajonc, 1965).

Following the insights on the global performance changes caused by the audience, the role of the spectators has been defined more precisely over the past decades. For example, Zajonc (1965) showed that when humans perform a simple or well-learned task, the presence of an audience increases the level of performance, while performance decreased for difficult or unfamiliar tasks. For professional soccer players, playing soccer is obviously a familiar task. Thus, according to the social facilitation theory, the presence of an audience may generally be perceived by them as facilitative or supportive rather than inhibitive or threatening.^1^ Perceptions of social support has been shown to increase athletes’ self-esteem and function as a buffer against stress (Freeman and Rees, 2010), to facilitate resilience (Galli and Vealey, 2008; Fletcher and Sarkar, 2012; Hill et al., 2018), and to be important for career success in soccer (Van Yperen, 2009). Interestingly, a meta-analysis showed that the home-field advantage was stronger for soccer than any other sport (Jamieson, 2010).

Following the restrictions that were implemented globally to combat the COVID-19 pandemic, spectators were prohibited from attending the matches played toward the end of 2019/2020 season. The absence of the spectators enabled researchers to study the effect of the crowd on the home field advantage in professional soccer across leagues and countries (e.g., Bryson et al., 2020; Cueva, 2020; Deutscher et al., 2020; Dilger and Vischer, 2020; Ferraresi and Gucciardi, 2020; Fischer and Haucap, 2020; Leitner and Richlan, 2020; McCarrick et al., 2020; Reade et al., 2020; Scoppa, 2020; Sors et al., 2020; Tilp and Thaller, 2020). There seems to be overall consensus that the absence of the crowd has a particularly strong effect in the German Bundesliga, even leading to several indicators for a home field *disadvantage* with only 44% (instead of more than 50%) of the total points being won by the home teams during this period (cf. Fischer and Haucap, 2020; Tilp and Thaller, 2020; Jiménez Sánchez and Lavín, 2021).

However, given that the available sample only reflects a portion of all matches from one season, these findings may be influenced by third variables. Although we can statistically control for various factors (e.g., difference in team strength), the outcomes of a dynamic sport such as soccer, can probably not be explained by isolated factors or their linear aggregates (cf. Kelso, 1995; Nowak and Vallacher, 1998; Van Orden et al., 2003; Den Hartigh et al., 2017; Hill et al., 2018). Instead, we can extract randomized subsamples with the same number of matches under the COVID-19 restrictions from the matches that haven been played previously in the same leagues to assess how frequently such relatively extreme outcomes as in Germany may be observed. This means that we can test whether the observed effects are due to chance. For example, if the same number of matches played during the COVID-19 restrictions is repeatedly sampled from previous years and these subsamples frequently show strong deviations from the typical home field advantage pattern, we may conclude that the effects are due to chance given the relatively small sample size. Conversely, if very few of these subsamples show results that are as extreme or more extreme, then the outcomes can confidently be attributed to the unique circumstances of the COVID-19 pandemic (e.g., the absence of spectators). This is because possible confounding factors, such as imbalances in team abilities, can be assumed to be distributed representatively across the subsamples when a sufficiently large number of subsamples is created.

An additional question of interest is which factors may explain the suspected loss of the home field advantage. In the vast literature on the home field advantage, several factors that may cause the home field advantage to emerge have been proposed. One prominent factor is the difference in the tactical approaches adopted by the teams when they play at home and away. According to Staufenbiel et al. (2015), coaches of home teams tend to decide for more offensive playing styles and challenging goals. In contrast, in the absence of a home crowd, the expectation of the away team’s coach to win may increase, which in turn, may cause the away teams to set approach-oriented and more challenging goals (Van Yperen, 2021). Such goals have repeatedly shown to cause better performance across various achievement contexts including sports (e.g., Van Yperen et al., 2015). Thus, a change in playing styles may be indicative of a change in the home field advantage.

Another frequently researched factor of the home field advantage is the so-called *referee bias* (e.g., Reade et al., 2020; Sors et al., 2020). The referee bias states that the referee favors the home team in their decision-making, by awarding proportionally more fouls, yellow cards, and red cards to the visitor’s side (Nevill et al., 2002; Boyko et al., 2007; Downward and Jones, 2007). However, because the referees are assumed to favor the home teams to avoid displeased reactions from the home crowd (Nevill et al., 2002), the bias may disappear when playing without spectators (Reade et al., 2020).

### The Current Study

The current study aims to test the changes in the home field advantage and its according match statistics as explanatory factors during the exclusion of spectators under COVID-19 (Bryson et al., 2020; Cueva, 2020; Deutscher et al., 2020; Dilger and Vischer, 2020; Ferraresi and Gucciardi, 2020; Fischer and Haucap, 2020; Leitner and Richlan, 2020; McCarrick et al., 2020; Reade et al., 2020; Scoppa, 2020; Tilp and Thaller, 2020; Jiménez Sánchez and Lavín, 2021). Specifically, because of the small sample sizes, we examined whether losing the home field advantage when playing behind closed doors is random rather than meaningful. To test this, we included professional soccer matches played in the four most highly ranked soccer leagues in Europe (i.e., the leagues that attract most audience): German Bundesliga, Spanish Primera Division, English Premier League, and Italian Serie A.^2^ Across the four leagues, and separately for each of these leagues, we created 1,000,000 random samples equal to the specific number of matches played without spectators from all matches played in the previous four full seasons before COVID-19 (i.e., 2015/2016 until 2018/2019). We hypothesized (*Hypothesis 1*) that relative to the random samples, under COVID-19 restrictions, the home field advantage would decline in terms of the proportion of points won by the home teams (e.g., Tilp and Thaller, 2020). To explain the suspected loss of the home field advantage, we also assessed indicators of offensive playing styles (i.e., goals, shots, and ball possession). We anticipated that these would decline among home teams and increase among away teams (*Hypothesis 2*; cf., Staufenbiel et al., 2015; Van Yperen, 2021). Furthermore, favorable referee decisions for the home team (i.e., fouls, yellow cards, and red cards) were hypothesized to disappear (*Hypothesis 3*; cf., Reade et al., 2020).

## Materials and Methods

### Procedure

For this study, we collected archival data from the German Bundesliga, the Spanish Primera Division, the English Premier League, and the Italian Serie A of the past five seasons (i.e., 2015/2016 until 2019/2020). Specifically, we obtained the final score, the number of shots from each team, and the ball possession distribution, as well as the number of fouls, yellow, and red cards awarded by the referee from each match that has been played during the regular season of this period. This means that relegation matches were not considered for the current study. Our total sample consisted of a total of 6,200 matches. Specifically, 5,784 matches were played over the previous four full seasons (i.e., for Italy, Spain, and England, 38 matchdays per season, 10 matches per matchday; for Germany, 34 matchdays per season, nine matches per matchday) and 416 matches under the COVID-19 restrictions (83 in Germany, 111 in Spain, 92 in England, and 130 in Italy). The data were accessed *via* the soccer statistics website www.transfermarkt.de between July 07, 2020 and August 28, 2020.^3^

### Measures

#### Points

Following Pollard (1986), we assessed the proportion of points won by the home teams from the total points earned in the played matches. In European soccer, three points are awarded to the team winning a match, with no points awarded to the losing team. If the game ends in a draw, each team receives one point. A home field advantage occurs when the home teams earn more than 50% of the total points distributed among the teams.

#### Offensive Team Statistics

Whereas the points indicate global match outcomes, goals, shots, and possession by both the home and away teams provide *a behavioral measure* of game performance. For example, goals do not only indicate the formal outcome of a match, but also *how* that outcome was achieved. Although all three measures are related (e.g., more shots increase the likelihood of scoring), they are distinct as, for example, different playing styles may result in different amounts of possession, but not necessarily in different numbers of shots. Similar to the proportional distribution of points (cf. Pollard, 1986), we determined the proportion of each offensive statistic by dividing the home teams’ score from the total number for each match. A home field advantage is associated with a proportion of goals scored, shots taken, and ball possession larger than 50%.

#### Referee Bias

In general, the referee bias describes the proportion of decisions made by the referee in favor of the home team (cf. Pollard, 1986). This includes the number fouls awarded as well as yellow and red cards given. These decisions may impact a game outcome because if a player receives a yellow card, they have to take less risk during physical challenges in order not to be sent off (making their team play short-handed for the rest of the match). Thus, if the referee gives more yellow cards to the away team, it may hinder their performance. Consequently, a home field advantage in referee decisions occurs when *fewer* than 50% of the decisions are against the home team.

### Data Analysis

For the analyses, we split the data into two separate periods: (1) baseline (i.e., the four full seasons preceding the COVID-19 pandemic 2015/16 until 2018/2019) and (2) matches from the 2019/2020 season without spectators due to the COVID-19 pandemic. To assess whether the changes in the outcomes and the associated statistics of the matches played without spectators is due randomness, we applied a Monte Carlo procedure (Van Geert et al., 2012). Specifically, we randomly selected the exact same number of matches played without spectators from all the matches in our sample that were played with spectators (i.e., baseline). For example, in Germany, 83 matches were played without spectators. For these randomly selected matches,^4^ we then determined the proportion of points won, the offensive statistics, and the referee bias for the home team. This procedure was repeated 1,000,000 times for all countries combined and each country individually to determine how many of the randomly created samples yielded a proportion that was as extreme or more extreme than the one observed during the matches played without spectators. This means that we counted how many randomly created samples showed the distribution of points and offensive statistics to be equal or smaller and indicators for referee bias to be larger than the observed values. If fewer than 50,000 (i.e., 5%) random samples yield equally or more extreme proportions, we can conclude that this effect is not due to randomness. This also increases our confidence that these outcomes are not due to potential, unobserved confounding variables.

## Results

First of all, we could replicate the home-field advantage as reported by Jamieson (2010). That is, more than 50% of the points were won by the home teams during the previous four full seasons between 2015/2016 and 2018/2019. Specifically, the home teams won 56.50% [55.73, 57.26] of all points across countries. For each country, the percentages were 56.68% [55.91, 57.44] in Germany, 57.27% [56.51, 58.03] in Spain, 56.27% [55.50, 57.03] in England, and 55.80% [55.04, 56.56] in Italy. Hence, our data consistently reconfirm that the largest proportion of the matches are won by the home teams, supporting the well-known home field advantage in soccer (Pollard, 1986; Jamieson, 2010).

Table 1 shows that across countries, all outcome variables, except for red cards, have changed beyond the level of chance to the disadvantage of the home team. However, Table 1 also indicates that this overall pattern could be ascribed to specific countries. Most importantly, we found empirical support for *Hypothesis 1* only in Germany. That is, only in Germany, the home field advantage turned into a disadvantage in terms of proportion of points won by the home teams (cf., Fischer and Haucap, 2020; Tilp and Thaller, 2020; Jiménez Sánchez and Lavín, 2021). As shown in Table 1, the proportion of points won by the home teams fell below 50% and only 0.10% (i.e., 1,000) of the 1,000,000 random samples showed equal or more extreme values. This latter percentage indicates that the loss of the home field advantage in Germany *was not* due to chance. In contrast, the COVID-19 restrictions did not kill the home field advantage in Spain, England, and Italy. That is, also under the COVID-19 restrictions, the proportion of points won by the home teams was higher than 50% and not significantly different from the proportions observed in the random samples.

**Table 1:** The results of the Monte Carlo permutation by country.

Country	Points		Goals		Shots		Possession		Fouls		Yellows		Reds	
	COVID	%	COVID	%	COVID	%	COVID	%	COVID	%	COVID	%	COVID	%
Overall	0.54	0.86^*^	0.53	1.99^*^	0.52	0.01^*^	0.51	2.73^*^	0.51	1.36^*^	0.52	0.01^*^	0.53	7.58
Germany	0.44	0.10^*^	0.46	0.14^*^	0.53	26.22	0.51	53.24	0.50	7.85	0.52	0.57^*^	0.53	28.03
Spain	0.55	10.12	0.54	13.63	0.52	0.20^*^	0.52	31.80	0.51	31.62	0.54	0.01^*^	0.57	9.55
England	0.58	46.77	0.57	56.04	0.52	1.81^*^	0.50	7.59	0.49	30.09	0.50	11.29	0.50	38.31
Italy	0.56	27.26	0.55	35.64	0.52	1.65^*^	0.50	1.18^*^	0.51	2.36^*^	0.51	0.09^*^	0.50	12.83

The value for the matches played without spectators is displayed under “COVID.” The percentage of the 1,000,000 random samples from the previous 5 years that yield a statistic that is as extreme or more extreme than the one observed is represented by “%.” *Values below 5% (i.e., chance level).

This loss of the home field advantage in Germany has been accompanied by significant changes in both offensive performance and referee decisions (see Table 1). Specifically, the proportion of goals scored by the home team fell below 50%, below the level of chance (0.14%), which was in line with *Hypothesis 2*. Similarly, some empirical support was obtained for *Hypothesis 3*: 52% of the yellow cards were given to the home teams, which exceeds the chance level of the random samples (0.57%). In contrast, the changes in the proportions of shots, possession, fouls, and red cards could not explain the loss of the home field advantage in Germany; the observed percentage were all within the boundaries of the random samples (see Table 1).

Despite the lack of significant changes in match outcomes, in the other three countries, the offensive playing performance also changed to the detriment of the home teams. Specifically, the proportion of shots decreased beyond chance in Spain (0.20%), England (1.81%), and Italy (1.65%), and only in Italy, the percentage of ball possession decreased (1.18%).

Furthermore, unrelated to match outcomes, under COVID-19 restrictions, referee decisions seem to favor the away teams in some countries. In terms of fouls, the distribution seems to be extreme only in Italy (2.36%) whereas the proportion of yellow cards given to the home teams is larger than chance in Spain (0.01%), and Italy (0.09%). Across the four countries, the proportion of the red cards is within the range of randomness, probably because relatively few red cards are issued per match.

## Discussion

Previous studies suggest that the absence of a home crowd changes the home field advantage in terms of match outcomes, offensive performance, and referee decisions. However, these studies typically relied on small sample sizes. Hence, these observed changes may be random rather than meaningful. In the present study, we tested this by creating 1,000,000 samples from the previous four seasons (2015/16 until 2018/2019) that were equal to the number of matches played under COVID-19 restrictions in Germany, Spain, England, and Italy at the end of the season 2019/2020. We found that across the four countries, performance indices and referee decisions (except red cards) indeed changed to the detriment of the home team beyond the level of chance. However, only in Germany, the home field advantage disappeared (cf. Fischer and Haucap, 2020; Tilp and Thaller, 2020; Jiménez Sánchez and Lavín, 2021), and this was not due to chance.

In recent studies, the most important explanation for the home field advantage in soccer that is beginning to surface is the refereeing bias towards the home team (Pollard and Gómez, 2014; Bryson et al., 2020; Cueva, 2020; Dilger and Vischer 2020; Endrich and Gesche, 2020; McCarrick et al., 2020; Reade et al., 2020; Scoppa, 2020; Tilp and Thaller 2020). For example, Reade et al. (2020) examined soccer matches played without spectators due to penalties for corruption or abusive behavior by the fans. They found that referees favored the home team less in their decision making when supporters were absent, which eroded the home field advantage. Their explanation was that referees may experience less pressure behind closed doors, and accordingly, make fewer calls in favor of the home teams, which in turn, results in more equal opportunities during the match for both the home and the away teams. Our results reconfirm that overall referee decisions tend to favor the away teams in matches played without spectators. This effect also holds across Germany, Spain, and Italy. However, despite these changes of the referee decisions, the match outcomes only changed in Germany. In other words, our data reconfirm that referees tend to favor the home team less in their decision-making when supporters were absent, but this does not necessarily erode the home field advantage.

Additionally, it should be noted that studies on the home field advantage typically lack actual behavioral indicators that are needed to test whether refereeing decisions are actually biased or not. Hence, in future studies, these behavioral indicators should be collected to test whether the “referee bias” actually represents biased decisions by referees (cf. Buraimo et al., 2010). An imbalance in the proportion of fouls or yellow and red cards does not necessarily represent biased decisions toward either the home or the away team, but accurate decisions to different behaviors displayed by the teams.

Furthermore, the implementation of the Video Assistant Referee (VAR) in Europe’s elite leagues aims at eliminating erroneous refereeing decisions during crucial match moments such as goals, penalties, or red cards. Therefore, the implementation of the VAR system in the 2019/2020 season may explain why we *did not* find a disappearance of the home field advantage in terms of match outcomes in Spain and Italy despite imbalanced decisions. That is, the referees’ tendencies to favor the home teams in crucial situations as an audience-effect may no longer exist in stadiums either with or without spectators through the reliance on the VAR.

Similar to the referee decisions, the tactical approaches adopted by the teams when playing at home and away tend to change when playing behind closed doors, but this also does not necessarily erode the home field advantage. A possible explanation for the loss of the home field advantage is that, in the absence of a home crowd, coaches may have lower expectations to win, set less challenging goals, and decide for less offensive and courageous playing tactics (Staufenbiel et al., 2015). In line with this reasoning, we found that across countries, the home teams played less offensively during the matches played under COVID-19 compared to the previous four seasons in terms of goals, shots, and possession. However, in Germany, where the home field advantage disappeared, we only observed a significant decline in the proportion of goals scored by the home team; there was no change for shots and ball possession. Reversely, the proportion of shots taken by home teams was unusually low in Spain, England, and Italy, and in Italy, there was even a decline in ball possession, but this was not accompanied by a loss of the home field advantage in terms of match outcomes. Thus, a lower proportion of points gained by the home team cannot unambiguously be explained by a less offensive playing style (Germany), and a less offensive playing style does not necessarily erase the home field advantage (e.g., Italy).

### Strengths, Limitations, and Future Directions

The key strength and unique contribution of the current article is that we have tested whether the observed changes in the home field advantage, offensive playing style, and referee bias under COVID-19 restrictions are meaningful or simply due to chance. By randomly selecting the same number of matches that were played under the COVID-19 restrictions from the previous four seasons 1,000,000 times, we statistically controlled for potential confounds, including teams’ performances during the previous seasons, imbalances in team abilities, quality of opposition, and competitive balance. These factors were assumed to be distributed equally and representatively across the huge number of subsamples that was created.

However, three potential limitations of the present study should be acknowledged. First and foremost, we do not have data to explore possible explanations for the observed differences between countries. Specifically, only in Germany, we found empirical evidence that the home field advantage disappeared during the COVID-19 pandemic. This means that although the safety protocols are rather similar across countries (including Germany, Italy, Spain, and England), the outcome of the behavioral adaptations in the German teams apparently differed from the other countries.

Secondly and relatedly, we do not have information what may have caused the decline of the home team performances. Although decreases in approach behavior and less offensive playing styles of the home teams are likely explanations, it is only partly supported by our data. While we observed a decrease in the proportion of shots by the home teams in Spain, England, and Italy, these leagues did not show a decline in proportion of points won. We hope that future studies will explore behavioral changes in more detail, including differences between leagues and countries. For example, future studies may consider assessing the changes in the tactical dynamics following the COVID-19 restrictions by studying centroid position data (Frencken et al., 2011) or the coordinated field position of individual players (Lames et al., 2010). In particular for the German league, where the strongest decline was found (i.e., the only country where the proportion of points dropped below 50%, cf., Deutscher et al., 2020; Fischer and Haucap, 2020; Tilp and Thaller, 2020), it may be argued that because the German league (May 16, 2020) resumed approximately 1 month prior to the Spanish (June 11, 2020), English (June 17, 2020), and Italian (June 20, 2020) league, the teams from the other countries had more time to prepare (e.g., tactically, and mentally) for the COVID-19 circumstances.

Finally, the observed outcomes of the matches played under the COVID-19 restrictions may not be due to the absence of the spectators alone. Specifically, the restrictions brought about several changes that may have had impact on the performances of the teams. For example, the strict protocols implemented by the soccer federations may have changed the manner in which especially the home teams prepared for the match and how they spent the nights leading up to the match. That is, in contrast to the normal situations, home team were quarantined in a hotel before a match. In contrast, for the away teams, staying in a hotel away from their family before the match is a rather common procedure. According to Jackson and Masters (2006), pre-game routines can help athletes focus on their task, which, in turn, prevent distractions that can disrupt their performance. Hence, a lack of usually applied rituals and routines for the home teams may have negatively impacted their performances. Another consequence of the COVID-19 restrictions is a crucial change of the rules by allowing for five substitutions compared to the usual three per match. This means that coaches had more opportunity to substitute tired players. This may be especially not only advantageous for the away teams to compensate for the strain experienced from traveling (cf. Oberhofer et al., 2010; Goumas, 2014; Leite and Pollard, 2018), but also for teams with a larger squad and more financial resources. However, if this is the case, the question remains why this would have affected the field home advantage only in Germany.

## Conclusion

In conclusion, the present study provides an empirical insight into the possible impact of the absence of spectators due to the COVID-19 restrictions on the performance of teams in European soccer leagues. We found that the proportion of points won by home teams substantially declined for the matches played without spectators in Germany. This change was meaningful rather than random and accompanied by changes in offensive performance (i.e., proportion of goals scored) and referee decisions (i.e., proportion of yellow cards). However, because offensive performances and referee decisions also changed disadvantageously for the home teams in the other three countries (except for referee decisions in England), the change in the match outcomes may not be attributable to these indicators. Overall, we conclude that the home field advantage may indeed be lost when spectators are absent, but more in-depth behavioral analyses, such as tactical dynamics, are needed to understand how the absence of spectators changes the game.

## Data Availability Statement

The original contributions presented in the study are included in the article/supplementary material, further inquiries can be directed to the corresponding author.

## Ethics Statement

Ethical review and approval was not required for the study on human participants in accordance with the local legislation and institutional requirements. Written informed consent for participation was not required for this study in accordance with the national legislation and the institutional requirements.

## Author Contributions

NY conceived the study. YH collected and analyzed the data. YH and NY wrote the manuscript. Both the authors contributed to the article and approved the submitted version.

### Conflict of Interest

The authors declare that the research was conducted in the absence of any commercial or financial relationships that could be construed as a potential conflict of interest.

## Footnotes

^1^Note that the same audience that is perceived as facilitative/supportive by the home team may be perceived as inhibitive/threatening by the away team. This may induce additional stress by excessively cheering for mistakes made by the players of the away team (cf. Jiménez Sánchez and Lavín, 2021) and, accordingly, add to the home field advantage effect as well.

^2^France’s first division also ranks among the top 5 leagues in Europe. However, in France, they did not continue their competition without spectators following the onset of the COVID-19 pandemic.

^3^Note that we compared the match scores obtained from this website with official websites from the national soccer associations (e.g., www.fussball.de for Germany).

^4^Any given match could only be selected once for a random sample. Thus, a random sample of 83 matches consisted of 83 unique matches without any repetitions.
